# Smoke Exposure Reduces the Protective Effect of Physical Activity on Hypertension: Evidence from the National Health and Nutrition Examination Survey (NHANES) 2013–2018

**DOI:** 10.3390/ijerph20032532

**Published:** 2023-01-31

**Authors:** Chen Li, Yiyi Wang, Shouyu Wang, Lei Huang

**Affiliations:** 1 Center for Public Health Research, Medical School of Nanjing University, Nanjing 210093, China; 2 State Key Laboratory of Pollution Control and Resource Reuse, School of the Environment, Nanjing University, Nanjing 210023, China; 3 Nanjing University (Suzhou) High-Tech Institute, Suzhou 215123, China

**Keywords:** serum cotinine concentrations, smoke exposure, physical activity, joint effects, hypertension, NHANES

## Abstract

The joint effects between smoke exposure (SE) and physical activity (PA) on hypertension are still unclear, and the effect of SE is still debated. To explore associations and joint effects of SE and PA on hypertension, the data of 14,456 selected participants from the NHANES (2013–2018) were used for analyses. SE status was divided by serum cotinine concentrations. Moderate-to-vigorous intensity PA (MVPA) and insufficient or no PA (INPA) were divided by the Global Physical Activity Questionnaire. Hypertension was assessed by blood pressure and questionnaires. Survey logistic multivariable regression models were conducted for data analyses. In fully adjusted models, hypertension risk among SE participants increased (OR = 1.175, 95% CI: 1.036–1.332), especially those who were <40 years or female. However, the risk among MVPA participants decreased (OR = 0.747, 95% CI: 0.663–0.841), especially those who were ≥40 years. Additionally, the OR for MVPA participants without SE when compared with INPA ones without SE was 0.740 (95% CI: 0.654–0.837), especially those who were <60 years. However, the OR for MVPA participants with SE was 0.880 (95% CI: 0.747–1.037). For INPA participants, we did not observe significant ORs for SE compared with non-SE participants (*p* > 0.150). In conclusion, SE increased the risk of hypertension and MVPA reduced it, but SE could reduce such protective effect.

## 1. Introduction

Hypertension and hazardously high blood pressure (BP) are responsible for 8.5 million deaths from cardiovascular disease and renal disease worldwide [1,2]. In 2019, the number of people aged 30–79 years with hypertension was 652 (95% confidence interval (CI): 604–698) million men and 626 (95% CI: 584–668) million women [3]. However, the rates of diagnosis, treatment, and control are all less than 50% globally [3,4]. Control and reducing the prevalence of hypertension is a major global health challenge and a primary public health issue. There are a lot of factors that put persons at risk for developing hypertension. Some of them are related to genetic and biological features, such as family history, age, race, and so on [5]. In addition, many behaviors in daily life can also increase the risk, and most of them are modifiable factors, including smoke exposure (SE), lack of physical activity (PA), stress, and high-sodium diet [5].

SE, including smoking and secondhand smoke exposure, is an essential environmental pollution problem nowadays, and is an independent risk factor for many diseases. For smoking, although it has declined over the past several years, about 19.0% of USA adults (47.1 million) reported using tobacco products, in 2020, such as cigarettes, e-cigarettes, etc. [6]. Some longitudinal studies have indicated that smoking is an independent risk factor for hypertension [7,8]. Additionally, the harm of secondhand smoke exposure is nearly as large as smoking [9]. However, the effect of SE on hypertension is still debated, some studies did not observe the significant negative associations between SE and hypertension [10,11].

*The Physical Activity Guidelines for Americans* recommends adults partake in at least 150 min of moderate-intensity PA or 75 min of vigorous-intensity PA a week, which could make them sleep, feel, and function better and reduce the risk of many chronic diseases [12]. Previous studies have pointed out that moderate-to-vigorous intensity PA (MVPA) could improve cardiorespiratory fitness and lower hypertension incidence [13]. Additionally, some professional organizations throughout the world recommend PA to lower BP [14].

Nowadays, many people not only have PA habits but also face SE in daily life. That is, they both have protective and risk factors for hypertension at the same time. However, current literature does not explore the joint effects of SE and PA on hypertension, which is an important evidence gap for preventing hypertension. Thus, in this study, we aim to explore the associations of SE and PA on hypertension, and the joint effects between them.

## 2. Materials and Methods

### 2.1. Study Participants

We used data, including demographic information, exposure assessments, outcome assessments, and confounders, from the National Health and Nutrition Examination Survey (NHANES) 2013–2018. The details about the NHANES are shown on the website of the Centers for Disease Control and Prevention of the USA (www.cdc.gov/nchs/nhanes/index.htm, accessed on 30 November 2022). In brief, the NHANES is a program of studies designed to assess the health and nutritional status of adults and children in the USA, which uses a cross-sectional, complex, multistage probability sample design to examine a nationally representative sample. In this study, we excluded participants who (1) were <20 years or recorded as 80 years; (2) did not complete the questionnaire; (3) did not have their serum cotinine concentration measured; and (4) could not determine whether participants had hypertension or not. At last, we included a total of 14,456 participants aged 20–79 years.

### 2.2. Exposure Assessments

#### 2.2.1. Smoke Exposure

The half-life of cotinine is about 16 h, making it a reliable biomarker of tobacco smoke exposure in both smokers and non-smokers [15]. In epidemiological studies, cotinine concentration is a better indicator of quantifying risks from smoking than a self-reported questionnaire, and serum is one of the fluids of choice for a quantitative assessment of such exposure [16,17]. On the other hand, cotinine is also a biomarker of secondhand smoke exposure [18,19]. Thus, we used serum cotinine level as the SE indicator instead of self-reported behavior. The detection method of serum cotinine concentration is provided in the Supplementary Materials. Based on a previous study on the USA population, 3.08 ng/mL of serum cotinine concentration was taken as the cut-off value to distinguish SE (≥3.08 ng/mL) from non-SE (<3.08 ng/mL) [17]. In this study, more than 30% of participants’ serum cotinine concentrations were below the limit of detection (LOD) (0.015 ng/mL), thus we used LOD/√2 (0.011 ng/mL) as a replacement.

#### 2.2.2. Physical Activity

The weekly PA habits of participants were collected by the Global Physical Activity Questionnaire (GPAQ). Briefly, the GPAQ was created by the World Health Organization (WHO) for collecting PA information in a typical week, including the time spent per week for PA in three domains (work, transportation, and recreation) at moderate and vigorous intensities (www.cdc.gov/nchs/nhanes/Default.aspx, accessed on 30 November 2022). According to the suggestions of *The Physical Activity Guidelines for Americans* and WHO, adults should partake in at least (1) 150 min/week of moderate-intensity PA, (2) 75 min/week of vigorous-intensity PA, or (3) an equivalent combination of PA achieving at 600 metabolic equivalent (MET)-minutes/week, including work, transport, or recreational activities [12,20]. In this study, we divided all the selected participants into two categories: (1) those with MVPA; (2) those with insufficient or no PA (INPA). We defined “MVPA” as participants with ≥600 MET-minutes/week, and others were defined as “INPA”. The relevant information about the GPAQ is provided in Table S1.

### 2.3. Outcome Assessments

BP, including systolic blood pressure (SBP) and diastolic blood pressure (DBP), was measured by BP examiners, who were certified for BP measurement through a training program from Shared Care Research and Education Consulting. Briefly, after resting quietly in a seated position for at least 5 min and after the participant’s maximum inflation level had been determined, three consecutive BP readings were obtained. The average of the three measurements of BP was calculated. Information about the self-reported hypertension was collected by questionnaires. We defined hypertension as a mean SBP ≥ 140 mmHg and/or a DBP ≥ 90 mmHg [21], self-reported use of anti-hypertensive medicine, or self-reporting himself/herself as a hypertension patient. The others were defined as healthy participants.

### 2.4. Confounders

**Figure 1:**
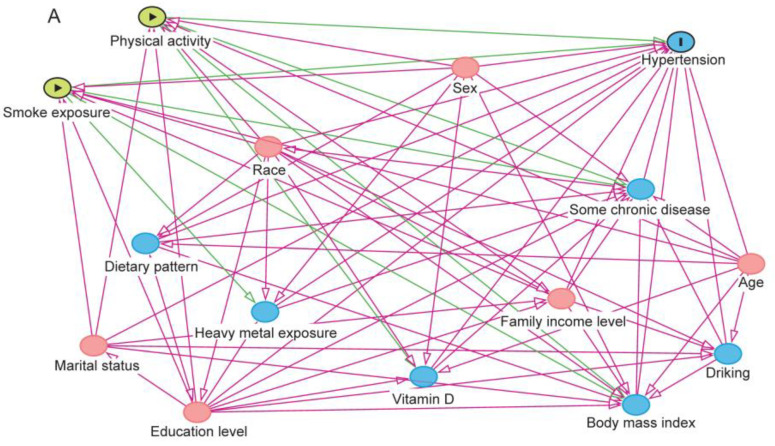

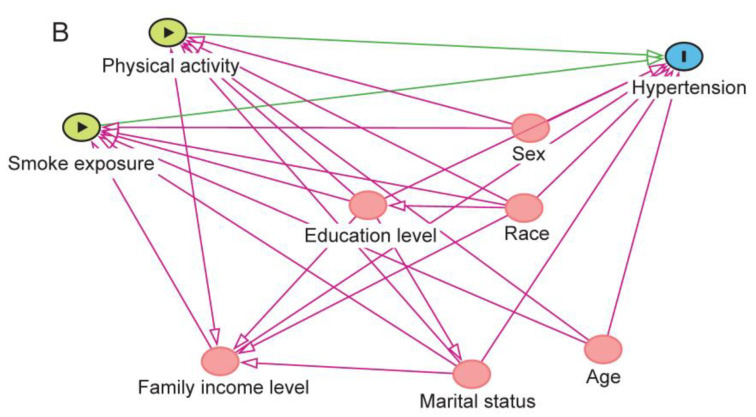
DAG for selecting confounders. (**A**) DAG for the associations among SE, PA, and hypertension, showing all potential confounders. (**B**) DAG for the associations among SE, PA, and hypertension, showing the confounders used in the final models. Pink lines indicate potential confounders, and green lines indicate potential mediators. Abbreviations: DAG, directed acyclic graph.

Firstly, we considered potential confounders according to the following criteria: (1) a risk factor for hypertension; (2) associated with SE or PA; (3) not be an “effect” of both two exposure assessments [22]. Then, we constructed a directed acyclic graph (DAG) with the help of DAGitty V3.0 (www.dagitty.net, accessed on 30 November 2022) [23,24] (Figure 1A). After that, we identified the following confounders based on the principle of the minimal sufficient adjustment sets: age (years), sex (male and female), race (Mexican American, other Hispanic, non-Hispanic White, non-Hispanic Black, and other race), educational level (<high school, high school graduate or equivalent, some college or AA degree, and >college graduate), marital status (living with spouse and living without spouse), and family poverty income ratio (PIR) (family PIR < 1, 1 ≤ family PIR < 2, 2 ≤ family PIR < 4, family PIR ≥ 4, and missing) [25] (Figure 1B). Additionally, we also included the survey wave as a confounder to control the potential differences among different waves.

### 2.5. Statistical Analyses

We used mean ± standard deviation (SD), median (interquartile range (IQR)), or *n* (%) to describe the different variables, including exposure and outcome assessments.

The NHANES used the complex and multistage probability sample design. Hence, we conducted survey logistic multivariable regression models to assess the associations of SE and PA with hypertension [26], using the R “survey” package, which is an analytical package for complex design survey data, such as NHANES (www.cran.r-project.org/web/packages/survey/index.html, accessed on 30 November 2022). Five levels of confounder adjustments were used: (1) crude models without any confounders (Model 1); (2) adjusted for age and sex in addition (Model 2); (3) adjusted for race, education level, marital status, and family PIR in addition (Model 3); (4) adjusted for survey wave in addition (Model 4); and (5) adjusted for another exposure assessment in addition (Model 5). The results were presented as odds ratios (ORs) for hypertension risk, and their corresponding 95% CI.

For evaluating the robustness of such results, we conducted several sensitivity analyses as follows. Model A: the participants without a family PIR (miss rate was 9.86%) were deleted. Model B: serum cotinine concentration was used as the exposure assessment instead of SE status. The other variables and statistical analysis methods of Model A and Model B were the same as Model 5.

Referring to previous studies [27,28,29], the joint effects of SE and PA on hypertension were examined after adjusting for all confounders (Model 5). First, we incorporated cross-product terms into regression models to identify interactions. Then, we divided each SE and PA variable into two groups. SE was divided into no and yes, and PA was divided into INPA and MVPA. We used the combinations of these categorical variables and classified them into the following four groups: non-SE with INPA (reference group), non-SE with MVPA, SE with INPA, and SE with MVPA.

All statistical analyses were performed in R V4.1.1 (R Foundation for Statistical Computing). A two-tailed *p* < 0.05 was considered as statistically significant.

## 3. Results

### 3.1. General Characteristics

A total of 14,456 participants were included from the NHANES with a mean age of 47.96 ± 16.28 years and the median (IQR) serum cotinine concentration was 0.04 (12.99) ng/mL. In this study, the weighted prevalence of hypertension was 37.42%, and the weighted proportions of SE participants and MVPA participants were 26.45% and 66.26%, respectively. The other general characteristics are shown in Table 1.

### 3.2. Associations of SE and PA on Hypertension

**Figure 2:**
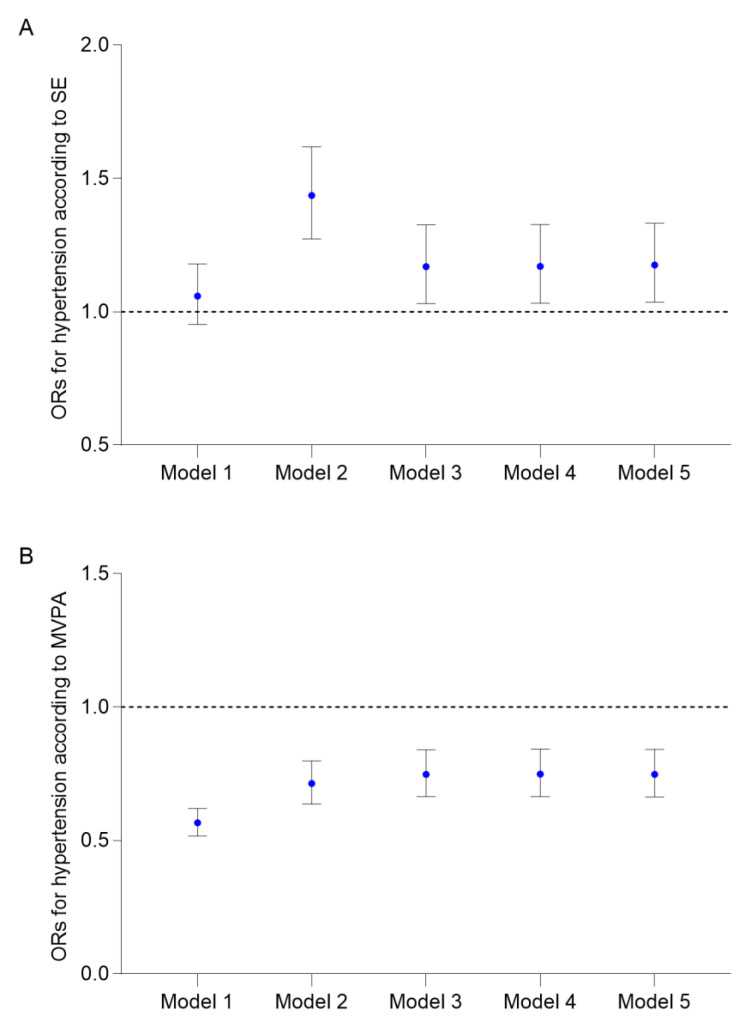
ORs for hypertension according to (**A**) SE (*n* = 14,456), (**B**) MVPA (*n* = 14,456). Model 1: crude model. Model 2: adjusted for age and sex. Model 3: adjusted for age, sex, race, education level, marital status, and family PIR. Model 4: adjusted for age, sex, race, education level, marital status, family PIR, and survey wave. Model 5: adjusted for age, sex, race, education level, marital status, family PIR, survey wave, and another exposure assessment. Abbreviations: ORs, odds ratios; other abbreviations as in Table 1.

Figure 2 and Table S2 present the results from the survey logistic multivariable regression analyses. In the fully adjusted model, the risk of hypertension for the participants with SE increased (OR = 1.175, 95% CI: 1.036–1.332), compared with those without SE. On the other hand, compared with participants who had INPA habits, participants who had MVPA habits had a lower risk of hypertension (OR = 0.747, 95% CI: 0.663–0.841).

### 3.3. Stratification Analyses by Age and Sex

**Figure 3:**
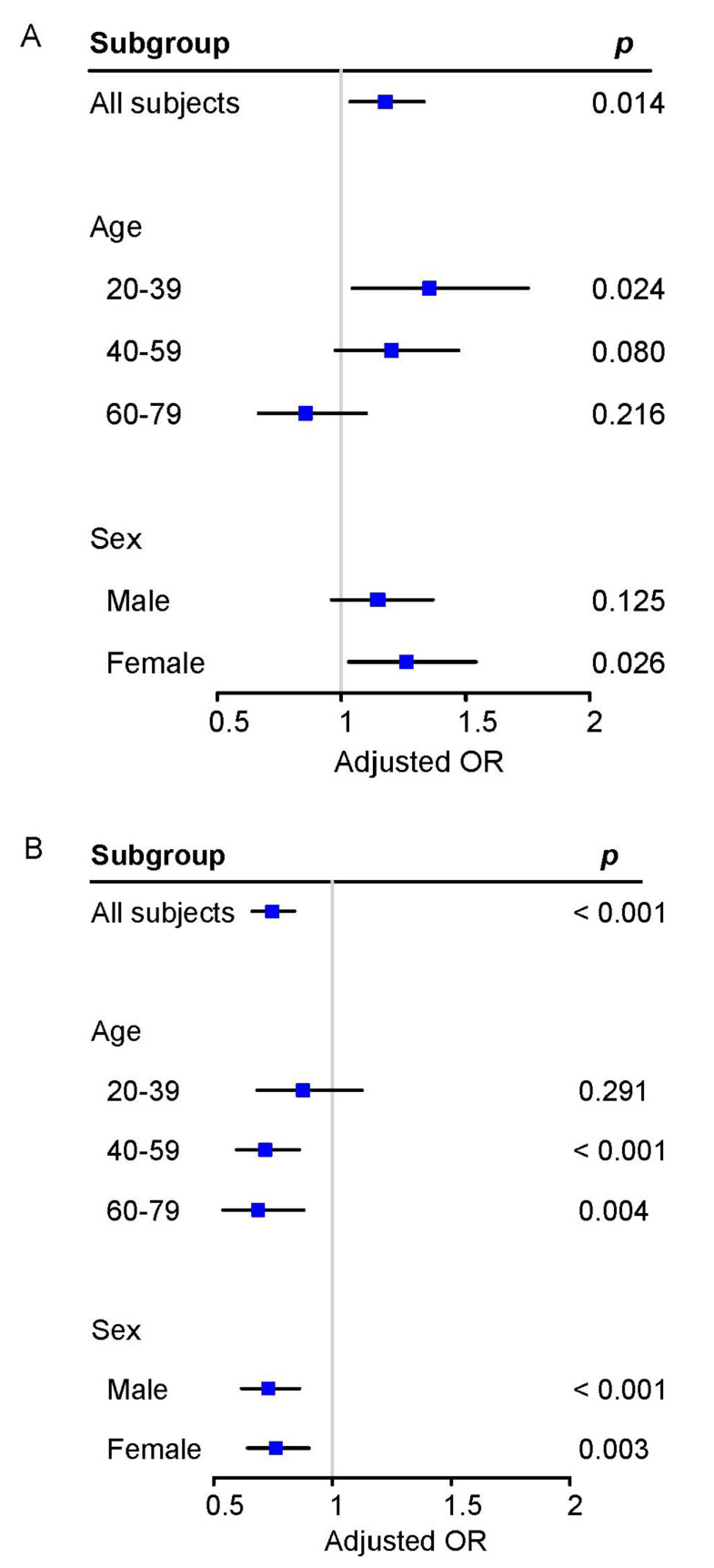
The results of stratification analyses by age and sex. (**A**) Associations between SE and hypertension stratified by age and sex (*n* = 14,456). (**B**) Associations between MVPA and hypertension stratified by age and sex (*n* = 14,456). Models were adjusted for age, sex, race, education level, marital status, family PIR, survey wave, and another exposure assessment. Abbreviations same as in Figure 2.

Tables S3–S7 show the general characteristics by different subgroups for age and sex. Meanwhile, Table 2 shows the weighted prevalence of hypertension and the weighted proportions of SE participants and MVPA participants for different subgroups. In stratified analyses by age, we found stronger associations between SE and hypertension among younger participants (<40 years), and stronger associations for MVPA among older participants (≥40 years) (Figure 3 and Table S8). In addition, we also found female participants had a significant association between SE and hypertension (OR = 1.260, 95% CI: 1.030–1.541), but no significance for male participants (*p* = 0.125) (Figure 3A and Table S8).

### 3.4. Sensitive Analyses

In sensitivity analyses, the results were also consistent with those from the main models (Model 5), when we deleted participants whose family PIR was missing or replaced SE status with serum cotinine concentrations (Table S9). That is, results from the main models had strong robustness.

### 3.5. Joint Effects between SE and PA on Hypertension

**Figure 4:**
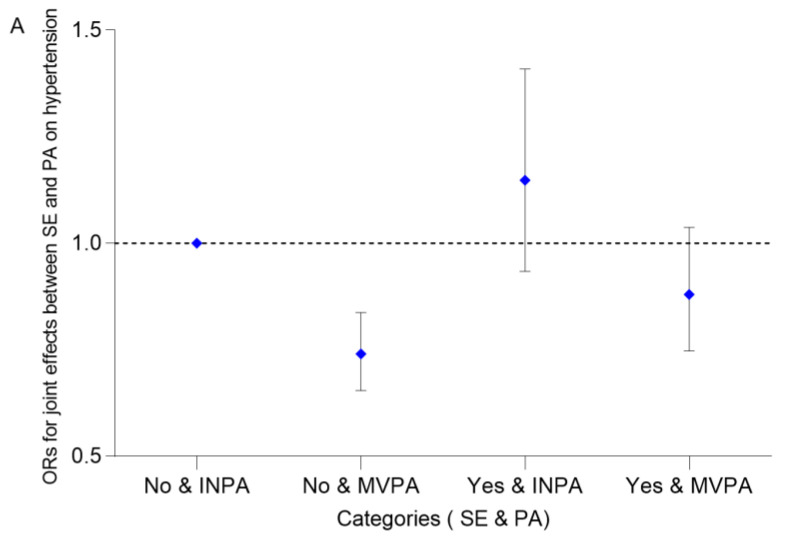

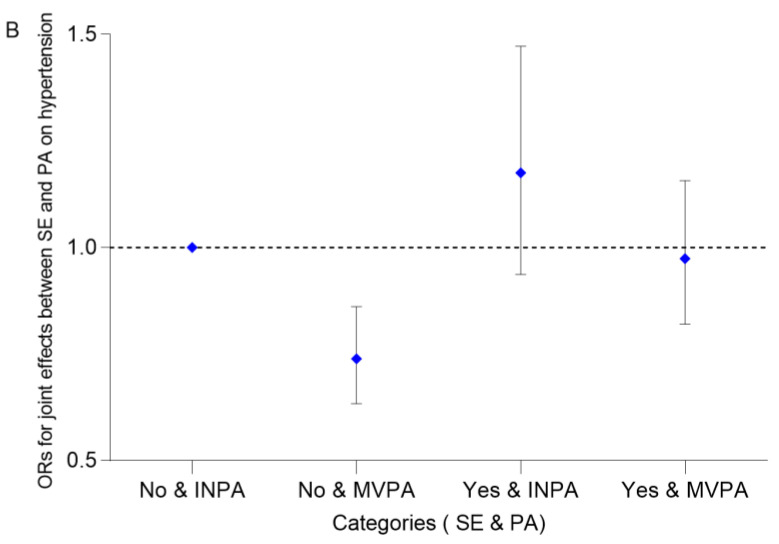
The joint effects between SE and PA on hypertension. (**A**) All participants (*n* = 14,456), *p* for interaction = 0.077. (**B**) Participants aged <60 years (*n* = 10,139), *p* for interaction = 0.043. Models were adjusted for age, sex, race, education level, marital status, family PIR, and survey wave. Abbreviations same as in Table 1 and Figure 2.

Figure 4 and Table S10 show the joint effects of SE and PA on hypertension. The OR for MVPA participants without SE when compared with the reference group was 0.740 (95% CI: 0.654–0.837). However, the OR for MVPA participants with SE was not significant (*p* = 0.122). In addition, the participants aged <60 years were the sensitive population (*p* for interaction = 0.043). General characteristics of this age group are shown in Table S11. Briefly, the weighted prevalence of hypertension was 28.16%, and the weighted proportions of SE participants and MVPA participants were 29.20% and 69.84%, respectively. Similar to the above results, the OR for MVPA participants without SE, when compared with the reference, was 0.739 (95% CI: 0.634–0.861). For INPA participants, we did not observe significant ORs for the participants with SE (*p* for all participants = 0.180, *p* for participants aged <60 years = 0.155).

## 4. Discussion

From our study, SE is a risk factor for hypertension, which increased the risk by 17.5% (95% CI: 3.6–33.2%), especially for those aged 20–39 years or female. While MVPA reduced the risk of hypertension by 25.3% (95% CI: 15.9–33.7%), and participants aged 40–79 years were the sensitive population. Moreover, we observed SE reduced the protective effect of PA on hypertension, as the OR for hypertension among participants who had MVPA habits without SE was 0.740 (95% CI: 0.654–0.837). Oppositely, we did not observe significant associations among participants with SE (*p* = 0.122). In addition, participants aged <60 years were the sensitive population for the joint effects (*p* for interaction = 0.043).

A 14-year longitudinal study of Japanese male workers indicated that the OR for smoking was 1.13 (95% CI: 1.03–1.23) for hypertension [8]. Additionally, a study based on the Korea Community Health Survey found smoking was significantly associated with hypertension risk (risk ratio (RR) = 1.016, 95% CI: 1.004–1.029), whilst secondhand smoke exposure at home (RR = 1.010, 95% CI: 1.006–1.014) or the workplace (RR = 1.004, 95% CI: 1.002–1.006) was also significantly associated [30]. An other study based on the NHANES database had shown that, compared with the serum cotinine concentrations of participants ≤ 0.025 ng/mL, the OR of hypertension among those with serum cotinine concentrations ≥ 0.218 ng/mL was 1.44 (95% CI: 1.01–2.04) [31]. Though SE has widely been considered an independent risk factor for hypertension, this conclusion is still inconsistent. A 5-year follow-up study among Japanese men found chronic smoking could reduce changes in BP and the 5-year cumulative incidence of hypertension [11]. Additionally, a Mendelian randomization study did not find a significant association between cigarettes per day and hypertension (*p* = 0.073) [32]. In our study, we found the OR for the association between SE and hypertension was 1.175 (95% CI: 1.036–1.332). These differences may be caused by study designs, exposure assessments, and population. Additionally, different subjects may consume different kinds of cigarettes and/or exposed to different concentrations of nicotine or other hazardous chemical substance. Meanwhile, the subjects of the above Japanese study were volunteers, which may cause more selection bias [11]. For different age and sex groups, the younger (20–39 years) or female population were more sensitive. An Iran study indicated a protective effect of smoking on hypertension (OR = 0.50, 95% CI: 0.41–0.60) among elderly persons (≥60 years) [33]; however, our research did not find a significant association among participants aged ≥60 years (*p* = 0.216). Similar to our results, a study from China Health and Nutrition Survey (CHNS) also did not find smoking as a significant risk factor for hypertension among male subjects (*p* > 0.05) [34]. Differences in genetic susceptibility or tolerance may be the reasons for the different results in different subgroups.

For PA, especially more than or equal to moderate-intensity is one of the lifestyles commonly recognized to reduce the risk of hypertension [12,35]. An interventional study, which was the first to explore the PA effects on reducing BP, found that an aerobic interval training program of 2 days per week could reduce BP among hypertension patients and normotensive men [36]. A review of 27 randomized controlled studies had shown that regular aerobic MVPA could reduce BP by a mean of 11/5 mmHg [37]. In addition to the daily activity, a longitudinal study according to the CHNS had also shown that moderate-intensity occupational PA is associated with a lower risk of new-onset hypertension [38]. Similarly, our study found that MVPA, including daily or occupational, was a protective factor against hypertension. However, there are also studies that did not find this protective effect of PA, such as a cohort study of young pre-hypertension patients, which did not find a significant association between PA and hypertension (*p* > 0.05) [39]. From our results, we also found that the middle-aged and elderly (40–79 years) may be the sensitive population, and the protective effect of PA was not found among the younger participants (20–39 years) (*p* = 0.291). Thus, these results indicate that the protective effect of PA on hypertension may differ among different age groups.

SE, mainly via the stimulation of the sympathetic nervous system, increases BP levels and the risk of hypertension [40]. In addition, SE can accelerate the atherothrombotic process because of the impairment of endothelial function, arterial stiffness, inflammation, etc., thus hypertension patients with SE are more likely to develop malignant and renovascular hypertension [40]. While an animal study suggested that one of the reasons for aerobic exercise reducing BP levels was improving the autonomic nervous system function [41]. Furthermore, PA could, through several other reasons, control BP levels, including reducing vascular resistance, inflammation level, and psychosocial stress, and increasing endothelial function, renal function, and angiogenesis [35]. From these insights, SE and PA have the same process to change BP levels and the risk of hypertension, which may be the reason why PA and SE have joint effects on it. From our results, compared with the INPA without SE and the MVPA without SE participants, hypertension risk reduced by 26.0% (95% CI: 16.3–34.6%). However, the risk effect of SE may be more serious than the protective effect of MVPA, and continual SE was more likely than continuing to exercise. These may be the reasons why our results found that SE could reduce the protective effects of PA.

To our knowledge, this is the first study to explore the joint effects between SE and PA on hypertension, which could provide reasonable suggestions for reducing hypertension. The NHANES is a national survey that examines a nationally representative sample, whose results are more persuasive by reflecting the overall situation in the USA. In addition to this, we used serum cotinine concentrations rather than self-reported questionnaires to assess the SE status, which is a better indicator to distinguish the SE status in epidemiological studies and could reduce information bias compared with self-reported questionnaires. We used survey logistic multivariable regression models to assess the associations between exposure assessments and outcome assessments. This is a better statistical method to process databases of the complex and multistage probability sample design, such as the NHANES.

However, our study also has several limitations. Firstly, the NHANES adopted a cross-sectional design, which precludes us from inferring any causal relationships. We cannot exclude reverse causality, though it is very unlikely. Secondly, the PA assessment was collected by self-reported questionnaires (GPAQ) according to memory and used MET scores to calculate the total MET value, thus the activity level of the participants may have information bias. Thirdly, the participants in this study were selected just from the USA, thus the results are not representative of other national populations, especially from developing countries. A longitudinal study with populations from more countries as well as a more detailed investigation should be considered in future.

## 5. Conclusions

The results of this study show that SE and PA both play important roles in the risk of hypertension, and the effect of SE may be more serious than the protective effect of MVPA on hypertension. All in all, MVPA can reduce the risk of hypertension but SE can increase it. In addition, SE can reduce this protective effect. Our findings suggest that the best way to enhance the protective effect of PA on hypertension is to avoid SE, including smoking and secondhand smoke exposure.

## Figures and Tables

**Table 1:** General characteristics of participants.

Variables	Overall (*n* = 14,456)	Hypertension or Not	
		No (*n* = 8444)	Yes (*n* = 6012)
Age (years), mean ± SD	47.96 ± 16.28	41.58 ± 14.88	56.94 ± 13.73
Sex, *n* (%)			
Male	6929 (47.93%)	3949 (56.99%)	2980 (43.01%)
Female	7527 (52.07%)	4495 (59.72%)	3032 (40.28%)
Serum cotinine concentration (ng/mL), median (IQR)	0.04 (12.99)	0.04 (10.89)	0.04 (19.04)
SE, *n* (%)			
No	10,542 (72.92%)	6150 (58.34%)	4392 (41.66%)
Yes	3914 (27.08%)	2294 (58.61%)	1620 (41.39%)
PA, *n* (%)			
INPA	5538 (38.31%)	2807 (50.69%)	2731 (49.31%)
MVPA	8918 (61.69%)	5637 (63.21%)	3281 (36.79%)
Race, *n* (%)			
Mexican American	2259 (15.63%)	1456 (64.45%)	803 (35.55%)
Other Hispanic	1586 (10.97%)	954 (60.15%)	632 (39.85%)
Non-Hispanic White	5072 (35.09%)	2992 (58.99%)	2080 (41.01%)
Non-Hispanic Black	3106 (21.49%)	1459 (46.97%)	1647 (53.03%)
Other race	2433 (16.83%)	1583 (65.06%)	850 (34.94%)
Education level, *n* (%)			
<High school	3074 (21.26%)	1658 (53.94%)	1416 (46.06%)
High school graduate or equivalent	3254 (22.51%)	1798 (55.26%)	1456 (44.74%)
Some college or AA degree	4531 (31.34%)	2627 (57.98%)	1904 (42.02%)
>College graduate	3597 (24.88%)	2361 (65.64%)	1236 (34.36%)
Marital status, *n* (%)			
Living without spouse	5664 (39.20%)	3225 (56.94%)	2439 (43.06%)
Living with spouse	8792 (60.80%)	5219 (59.36%)	3573 (40.64%)
Family PIR, *n* (%)			
<1.0	2786 (19.27%)	1592 (57.14%)	1194 (42.86%)
1.0–2.0	3474 (24.03%)	1970 (56.71%)	1504 (43.29%)
2.0–4.0	3460 (23.93%)	2062 (59.60%)	1398 (40.40%)
≥4.0	3311 (22.90%)	2016 (60.89%)	1295 (39.11%)
Missing	1425 (9.86%)	804 (56.42%)	621 (43.58%)

Abbreviations: SD, standard deviations; IQR, interquartile range; SE, smoke exposure; PA, physical activity; INPA, insufficient or no physical activity; MVPA, moderate-to-vigorous intensity physical activity; PIR, poverty income ratio.

**Table 2:** Weighted prevalence of hypertension, and proportions of SE participants and MVPA participants among the different subgroups.

Subgroup	*n*	Weighted Prevalence/Proportions		
		Hypertension	SE	MVPA
Age				
20–39	5018	15.90%	31.22%	75.78%
40–59	5121	40.39%	27.19%	63.92%
60–79	4317	65.65%	18.05%	55.35%
Sex				
Male	6929	39.28%	31.77%	72.47%
Female	7527	35.67%	21.42%	60.40%

Abbreviations same as in Table 1.

## Data Availability

All data of this research were collected from the NHANES (www.cdc.gov/nchs/nhanes/index.htm, accessed on 30 November 2022).

## Supplementary Materials

The following supporting information can be downloaded at: https://www.mdpi.com/article/10.3390/ijerph20032532/s1. Table S1. The texts of Global Physical Activity Questionnaire related to this study. Table S2. ORs for hypertension according to SE and MVPA. Table S3. General characteristics of participants aged 20–39 years. Table S4. General characteristics of participants aged 40–59 years. Table S5. General characteristics of participants aged 60–79 years. Table S6. General characteristics of male participants. Table S7. General characteristics of female participants. Table S8. ORs for hypertension according to SE and MVPA by age and sex. Table S9. The results of sensitive analyses. Table S10. Joint effects between SE and PA on hypertension. Table S11. General characteristics of participants aged less than 60 years.

## References

[1] Zhou B., Perel P., Mensah G.A., Ezzati M. (2021). Global epidemiology, health burden and effective interventions for elevated blood pressure and hypertension. Nat. Rev. Cardiol..

[2] Olsen M.H., Angell S.Y., Asma S., Boutouyrie P., Burger D., Chirinos J.A., Damasceno A., Delles C., Gimenez-Roqueplo A.P., Hering D. (2016). A call to action and a lifecourse strategy to address the global burden of raised blood pressure on current and future generations: The Lancet Commission on hypertension. Lancet.

[3] NCD Risk Factor Collaboration (NCD-RisC) (2021). Worldwide trends in hypertension prevalence and progress in treatment and control from 1990 to 2019: A pooled analysis of 1201 population-representative studies with 104 million participants. Lancet.

[4] Mills K.T., Bundy J.D., Kelly T.N., Reed J.E., Kearney P.M., Reynolds K., Chen J., He J. (2016). Global Disparities of Hypertension Prevalence and Control: A Systematic Analysis of Population-Based Studies From 90 Countries. Circulation.

[5] Know Your Risk Factors for High Blood Pressure. https://www.heart.org/en/health-topics/high-blood-pressure/why-high-blood-pressure-is-a-silent-killer/know-your-risk-factors-for-high-blood-pressure.

[6] Cornelius M.E., Loretan C.G., Wang T.W., Jamal A., Homa D.M. (2022). Tobacco product use among adults—United States, 2020. MMWR Morb. Mortal. Wkly. Rep..

[7] Niskanen L., Laaksonen D.E., Nyyssonen K., Punnonen K., Valkonen V.P., Fuentes R., Tuomainen T.P., Salonen R., Salonen J.T. (2004). Inflammation, abdominal obesity, and smoking as predictors of hypertension. Hypertension.

[8] Dochi M., Sakata K., Oishi M., Tanaka K., Kobayashi E., Suwazono Y. (2009). Smoking as an independent risk factor for hypertension: A 14-year longitudinal study in male Japanese workers. Tohoku J. Exp. Med..

[9] Skipina T.M., Soliman E.Z., Upadhya B. (2020). Association between secondhand smoke exposure and hypertension: Nearly as large as smoking. J. Hypertens..

[10] Sohn K. (2018). Relationship of Smoking to Hypertension in a Developing Country. Glob. Heart.

[11] Okubo Y., Suwazono Y., Kobayashi E., Nogawa K. (2004). An association between smoking habits and blood pressure in normotensive Japanese men: A 5-year follow-up study. Drug Alcohol Depend..

[12] Piercy K.L., Troiano R.P., Ballard R.M., Carlson S.A., Fulton J.E., Galuska D.A., George S.M., Olson R.D. (2018). The Physical Activity Guidelines for Americans. JAMA.

[13] Bakker E.A., Sui X., Brellenthin A.G., Lee D.C. (2018). Physical activity and fitness for the prevention of hypertension. Curr. Opin. Cardiol..

[14] Pescatello L.S., MacDonald H.V., Ash G.I., Lamberti L.M., Farquhar W.B., Arena R., Johnson B.T. (2015). Assessing the Existing Professional Exercise Recommendations for Hypertension: A Review and Recommendations for Future Research Priorities. Mayo Clin. Proc..

[15] Benowitz N.L., Jacob P. (1994). Metabolism of nicotine to cotinine studied by a dual stable isotope method. Clin. Pharmacol. Ther..

[16] Perezstable E.J., Benowitz N.L., Marin G. (1995). Is Serum Cotinine a Better Measure of Cigarette-Smoking Than Self-Report. Prev. Med..

[17] Benowitz N.L., Bernert J.T., Caraballo R.S., Holiday D.B., Wang J.T. (2009). Optimal serum cotinine levels for distinguishing cigarette smokers and nonsmokers within different racial/ethnic groups in the United States between 1999 and 2004. Am. J. Epidemiol..

[18] Sim B., Park M.B. (2021). Exposure to Secondhand Smoke: Inconsistency between Self-Response and Urine Cotinine Biomarker Based on Korean National Data during 2009–2018. Int. J. Environ. Res. Public Health.

[19] Zhang Z.M., Li Z.P., Zhang X.Y., Ye W.Y., Chen J.Q., Wang L., Lin Z.L., Li J., Li Z.H. (2022). Association between secondhand smoke and cancers in adults in the US population. J. Cancer Res. Clin. Oncol..

[20] Physical Activity. https://www.who.int/news-room/fact-sheets/detail/physical-activity.

[21] DeGuire J., Clarke J., Rouleau K., Roy J., Bushnik T. (2019). Blood pressure and hypertension. Health Rep..

[22] Jager K.J., Zoccali C., Macleod A., Dekker F.W. (2008). Confounding: What it is and how to deal with it. Kidney Int..

[23] Greenland S., Pearl J., Robins J.M. (1999). Causal diagrams for epidemiologic research. Epidemiology.

[24] Williamson E.J., Aitken Z., Lawrie J., Dharmage S.C., Burgess J.A., Forbes A.B. (2014). Introduction to causal diagrams for confounder selection. Respirology.

[25] Hoge C., Bowling C.B., Lim S.S., Drenkard C., Plantinga L.C. (2020). Association of Poverty Income Ratio with Physical Functioning in a Cohort of Patients with Systemic Lupus Erythematosus. J. Rheumatol..

[26] Lumley T., Scott A. (2017). Fitting Regression Models to Survey Data. Stat. Sci..

[27] Gao X., Huang N., Guo X., Huang T. (2022). Role of sleep quality in the acceleration of biological aging and its potential for preventive interaction on air pollution insults: Findings from the UK Biobank cohort. Aging Cell.

[28] Min J.E., Huh D.A., Moon K.W. (2020). The Joint Effects of Some Beverages Intake and Smoking on Chronic Obstructive Pulmonary Disease in Korean Adults: Data Analysis of the Korea National Health and Nutrition Examination Survey (KNHANES), 2008–2015. Int. J. Environ. Res. Public Health.

[29] Zuk A.M., Quinonez C.R., Saarela O., Demmer R.T., Rosella L.C. (2018). Joint effects of serum vitamin D insufficiency and periodontitis on insulin resistance, pre-diabetes, and type 2 diabetes: Results from the National Health and Nutrition Examination Survey (NHANES) 2009–2010. BMJ Open Diabetes Res. Care.

[30] Lee W., Hwang S.H., Choi H., Kim H. (2017). The association between smoking or passive smoking and cardiovascular diseases using a Bayesian hierarchical model: Based on the 2008–2013 Korea Community Health Survey. Epidemiol. Health.

[31] Alshaarawy O., Xiao J., Shankar A. (2013). Association of serum cotinine levels and hypertension in never smokers. Hypertension.

[32] Van Oort S., Beulens J.W.J., van Ballegooijen A.J., Grobbee D.E., Larsson S.C. (2020). Association of Cardiovascular Risk Factors and Lifestyle Behaviors with Hypertension: A Mendelian Randomization Study. Hypertension.

[33] Mehboudi M., Nabipour I., Vahdat K., Darabi H., Raeisi A., Mehrdad N., Heshmat R., Shafiee G., Larijani B., Ostovar A. (2017). Inverse association between cigarette and water pipe smoking and hypertension in an elderly population in Iran: Bushehr elderly health programme. J. Hum. Hypertens..

[34] Li G., Wang H., Wang K., Wang W., Dong F., Qian Y., Gong H., Hui C., Xu G., Li Y. (2017). The association between smoking and blood pressure in men: A cross-sectional study. BMC Public Health.

[35] Diaz K.M., Shimbo D. (2013). Physical activity and the prevention of hypertension. Curr. Hypertens. Rep..

[36] Boyer J.L., Kasch F.W. (1970). Exercise therapy in hypertensive men. JAMA.

[37] Borjesson M., Onerup A., Lundqvist S., Dahlof B. (2016). Physical activity and exercise lower blood pressure in individuals with hypertension: Narrative review of 27 RCTs. Br. J. Sports Med..

[38] Li Q., Li R., Zhang S., Zhang Y., He P., Zhang Z., Liu M., Zhou C., Li H., Liu C. (2021). Occupational Physical Activity and New-Onset Hypertension: A Nationwide Cohort Study in China. Hypertension.

[39] Lu Y., Lu M., Dai H., Yang P., Smith-Gagen J., Miao R., Zhong H., Chen R., Liu X., Huang Z. (2015). Lifestyle and Risk of Hypertension: Follow-Up of a Young Pre-Hypertensive Cohort. Int. J. Med. Sci..

[40] Virdis A., Giannarelli C., Neves M.F., Taddei S., Ghiadoni L. (2010). Cigarette smoking and hypertension. Curr. Pharm. Des..

[41] Moraes-Silva I.C., Mostarda C., Moreira E.D., Silva K.A., dos Santos F., de Angelis K., Farah Vde M., Irigoyen M.C. (2013). Preventive role of exercise training in autonomic, hemodynamic, and metabolic parameters in rats under high risk of metabolic syndrome development. J. Appl. Physiol..

