# Supplementary material for: Smoke Exposure Reduces the Protective Effect of Physical Activity on Hypertension: Evidence from the National Health and Nutrition Examination Survey (NHANES) 2013–2018
Source: Int J Environ Res Public Health. 2023 Jan 31;20(3):2532. doi: 10.3390/ijerph20032532 (PMC9915007; doi:10.3390/ijerph20032532)
Supplement: Supplementary file 1 [file ijerph-20-02532-s001.zip › ijerph-2104408-supplementary.pdf]

Smoke Exposure Reduces the Protective Effect of Physical Activity on Hypertension: Evidence from the National Health and Nutrition Examination Survey (NHANES) 2013-2018

Supplementary Materials

Supplementary Methods

Detection Method of Serum Cotinine Concentration Page 2

Supplementary Tables

Table S1. The texts of Global Physical Activity Questionnaire related to this study. Page 3

Table S2. ORs for hypertension according to SE and MVPA. Page 5

Table S3. General characteristics of participants aged 20-39 years. Page 6

Table S4. General characteristics of participants aged 40-59 years. Page 7

Table S5. General characteristics of participants aged 60-79 years. Page 8

Table S6. General characteristics of male participants. Page 9

Table S7. General characteristics of female participants. Page 10

Table S8. ORs for hypertension according to SE and MVPA by age and sex. Page 11

Table S9. The results of sensitive analyses. Page 12

Table S10. Joint effects between SE and PA on hypertension. Page 13

Table S11. General characteristics of participants aged less than 60 years. Page 14

Supplementary Methods

Detection Method of Serum Cotinine Concentration

Serum cotinine was measured by an isotope-dilution high-performance liquid chromatography/atmospheric pressure chemical ionization tandem mass spectrometric (ID HPLC-APCI MS/MS) method. Briefly, the serum sample is spiked with methyl-D3-cotinine and methyl-D3-hydroxycotinine as internal standards. The sample is basified and then applied to a supported liquid extraction plate. The analytes are extracted with an isopropanol/methylene chloride mixture, the organic extract is concentrated, and the residue is injected onto a C18 HPLC column. The eluent from these injections is monitored by APCI-MS/MS. The m/z 80 product ion from the m/z 177 quasi-molecular ion is measured for cotinine. Additional ions for the internal standards and for confirmation are also monitored for the respective compounds. Analyte concentrations are derived from the area ratios of native-to-labeled compounds in the sample by comparisons to a standard curve.

The above brief method was excerpted from the NHANES (wwwn.cdc.gov/nchs/nhanes/Default.aspx, accessed on 30 November 2022), and detailed methods also could be found from this website.

Supplementary Tables

Table S1. The texts of Global Physical Activity Questionnaire related to this study.

Items	Variable name	English Text	Suggested MET Scores
Vigorous-intensity work activity	PAQ605	Next I am going to ask you about the time {you spend/SP spends} doing different types of physical activity in a typical week. Think first about the time {you spend/he spends/she spends} doing work. Think of work as the things that {you have/he has/she has} to do such as paid or unpaid work, household chores, and yard work. Does {your/SP's} work involve vigorous-intensity activity that causes large increases in breathing or heart rate like carrying or lifting heavy loads, digging or construction work for at least 10 minutes continuously?	8.0
	PAQ610	In a typical week, on how many days {do you/does SP} do vigorous-intensity activities as part of {your/his/her} work?	
	PAQ615	How much time {do you/does SP} spend doing vigorous-intensity activities at work on a typical day?	
Moderate-intensity work activity	PAQ620	Does {your/SP's} work involve moderate-intensity activity that causes small increases in breathing or heart rate such as brisk walking or carrying light loads for at least 10 minutes continuously?	4.0
	PAQ625	In a typical week, on how many days {do you/does SP} do moderate-intensity activities as part of {your/his/her} work?	
	PAQ630	How much time {do you/does SP} spend doing moderate-intensity activities at work on a typical day?	
Transport activities (walk or bicycle)	PAQ635	The next questions exclude the physical activity at work that you have already mentioned. Now I would like to ask you about the usual way {you travel/SP travels} to and from places. For example to school, for shopping, to work. In a typical week {do you/does SP} walk or use a bicycle for at least 10 minutes continuously to get to and from places?	4.0

	PAQ640	In a typical week, on how many days {do you/does SP} walk or bicycle for at least 10 minutes continuously to get to and from places?	
	PAQ645	How much time {do you/does SP} spend walking or bicycling for travel on a typical day?	
Vigorous-intensity recreational activities	PAQ650	The next questions exclude the work and transport activities that you have already mentioned. Now I would like to ask you about sports, fitness and recreational activities. In a typical week {do you/does SP} do any vigorous-intensity sports, fitness, or recreational activities that cause large increases in breathing or heart rate like running or basketball for at least 10 minutes continuously?	8.0
	PAQ655	In a typical week, on how many days {do you/does SP} do vigorous-intensity sports, fitness or recreational activities?	
	PAQ660	How much time {do you/does SP} spend doing vigorous-intensity sports, fitness or recreational activities on a typical day?	
Moderate-intensity recreational activities	PAQ665	In a typical week {do you/does SP} do any moderate-intensity sports, fitness, or recreational activities that cause a small increase in breathing or heart rate such as brisk walking, bicycling, swimming, or volleyball for at least 10 minutes continuously?	4.0
	PAQ670	In a typical week, on how many days {do you/does SP} do moderate-intensity sports, fitness or recreational activities?	
	PAQ675	How much time {do you/does SP} spend doing moderate-intensity sports, fitness or recreational activities on a typical day?	

Notes: These texts were excerpted from the NHANES (wwwn.cdc.gov/nchs/nhanes/Default.aspx, accessed on 30 November 2022). Abbreviations: SP, sampled person; MET, metabolic equivalent.

Table S2. ORs for hypertension according to SE and MVPA. (n = 14456)

Variables	β	Wald	OR	95% CI	p
SE					
Model 1	0.057	1.168	1.059	0.952-1.179	0.286
Model 2	0.362	36.955	1.436	1.273-1.619	**< 0.001**
Model 3	0.156	6.435	1.169	1.031-1.326	**0.017**
Model 4	0.157	6.573	1.170	1.032-1.327	**0.016**
Model 5	0.161	6.874	1.175	1.036-1.332	**0.014**
MVPA					
Model 1	-0.569	157.733	0.566	0.516-0.620	**< 0.001**
Model 2	-0.339	36.206	0.713	0.636-0.798	**< 0.001**
Model 3	-0.291	25.636	0.747	0.664-0.840	**< 0.001**
Model 4	-0.290	25.250	0.748	0.664-0.842	**< 0.001**
Model 5	-0.292	25.457	0.747	0.663-0.841	**< 0.001**

Notes: Model 1: crude model. Model 2: adjusted for age and sex. Model 3: adjusted for age, sex, race, education level, marital status, family PIR. Model 4: adjusted for age, sex, race, education level, marital status, family PIR, and survey wave. Model 5: adjusted for age, sex, race, education level, marital status, family PIR, survey wave, and another exposure assessment. Abbreviations: SE, smoke exposure; MVPA, moderate-to-vigorous intensity physical activity; OR, odds ratio; CI, confidence interval; PIR, poverty income ratio.

Table S3. General characteristics of participants aged 20-39 years.

**Variables**	**Overall** (*n* = 5018)	**Hypertension or not**	
		No (*n* = 4223)	Yes (*n* = 795)
Age (years), mean ± SD	29.57 ± 5.68	29.14 ± 5.64	31.86 ± 5.31
Sex, *n* (%)			
Male	2390 (47.63%)	1935 (80.96%)	455 (19.04%)
Female	2628 (52.37%)	2288 (87.06%)	340 (12.94%)
SE, *n* (%)			
No	3405 (67.86%)	2927 (85.96%)	478 (14.04%)
Yes	1613 (32.14%)	1296 (80.35%)	317 (19.65%)
PA, *n* (%)			
INPA	1362 (27.14%)	1143 (83.92%)	219 (16.08%)
MVPA	3656 (72.86%)	3080 (84.25%)	576 (15.75%)
Race, *n* (%)			
Mexican American	824 (16.42%)	714 (86.65%)	110 (13.35%)
Other Hispanic	517 (10.30%)	465 (89.94%)	52 (10.06%)
Non-Hispanic White	1725 (34.38%)	1430 (82.90%)	295 (17.10%)
Non-Hispanic Black	1026 (20.45%)	814 (79.34%)	212 (20.66%)
Other race	926 (18.45%)	800 (86.39%)	126 (13.61%)
Education level, *n* (%)			
< high school	844 (16.82%)	708 (83.89%)	136 (16.11%)
High school graduate or equivalent	1130 (22.52%)	937 (82.92%)	193 (17.08%)
Some college or AA degree	1768 (35.23%)	1466 (82.92%)	302 (17.08%)
> college graduate	1276 (25.43%)	1112 (87.15%)	164 (12.85%)
Marital status, *n* (%)			
Living without spouse	2272 (45.28%)	1928 (84.86%)	344 (15.14%)
Living with spouse	2746 (54.72%)	2295 (83.58%)	451 (16.42%)
Family PIR, *n* (%)			
< 1.0	1120 (22.32%)	932 (83.21%)	188 (16.79%)
1.0-2.0	1235 (24.61%)	1020 (82.59%)	215 (17.41%)
2.0-4.0	1233 (24.57%)	1043 (84.59%)	190 (15.41%)
≥ 4.0	972 (19.37%)	836 (86.01%)	136 (13.99%)
missing	458 (9.13%)	392 (85.59%)	66 (14.41%)

Abbreviations: SD, standard deviation; PA, physical activity; INPA, insufficient or no physical activity; other abbreviations as in Table S2.

Table S4. General characteristics of participants aged 40-59 years.

Variables	Overall (n = 5121)	Hypertension or not	
		No (n = 2935)	Yes (n = 2186)
Age (years), mean ± SD	49.44 ± 5.76	48.42 ± 5.63	50.82 ± 5.64
Sex, n (%)			
Male	2392 (46.71%)	1334 (55.77%)	1058 (44.23%)
Female	2729 (53.29%)	1601 (58.67%)	1128 (41.33%)
SE, n (%)			
No	3696 (72.17%)	2202 (59.58%)	1494 (40.42%)
Yes	1425 (27.83%)	733 (51.44%)	692 (48.56%)
PA, n (%)			
INPA	2024 (39.52%)	1090 (53.85%)	934 (46.15%)
MVPA	3097 (60.48%)	1845 (59.57%)	1252 (40.43%)
Race, n (%)			
Mexican American	795 (15.52%)	527 (66.29%)	268 (33.71%)
Other Hispanic	546 (10.66%)	316 (57.88%)	230 (42.12%)
Non-Hispanic White	1726 (33.70%)	1016 (58.86%)	710 (41.14%)
Non-Hispanic Black	1102 (21.52%)	461 (41.83%)	641 (58.17%)
Other race	952 (18.59%)	615 (64.60%)	337 (35.40%)
Education level, n (%)			
< high school	1080 (21.09%)	622 (57.59%)	458 (42.41%)
High school graduate or equivalent	1113 (21.73%)	590 (53.01%)	523 (46.99%)
Some college or AA degree	1555 (30.37%)	823 (52.93%)	732 (47.07%)
> college graduate	1373 (26.81%)	900 (65.55%)	473 (34.45%)
Marital status, n (%)			
Living without spouse	1671 (32.63%)	841 (50.33%)	830 (49.67%)
Living with spouse	3450 (67.37%)	2094 (60.70%)	1356 (39.30%)
Family PIR, n (%)			
< 1.0	911 (17.79%)	475 (52.14%)	436 (47.86%)
1.0-2.0	1105 (21.58%)	632 (57.19%)	473 (42.81%)
2.0-4.0	1223 (23.88%)	718 (58.71%)	505 (41.29%)
≥ 4.0	1390 (27.14%)	838 (60.29%)	552 (39.71%)
missing	492 (9.61%)	272 (55.28%)	220 (44.72%)

Abbreviations as in Table S3.

Table S5. General characteristics of participants aged 60-79 years.

Variables	Overall (*n* = 4317)	Hypertension or not	
		No (*n* = 1286)	Yes (*n* = 3031)
Age (years), mean ± SD	67.59 ± 5.49	66.78 ± 5.27	67.93 ± 5.55
Sex, *n* (%)			
Male	2147 (49.73%)	680 (31.67%)	1467 (68.33%)
Female	2170 (50.27%)	606 (27.93%)	1564 (72.07%)
SE, *n* (%)			
No	3441 (79.71%)	1021 (29.67%)	2420 (70.33%)
Yes	876 (20.29%)	265 (30.25%)	611 (69.75%)
PA, *n* (%)			
INPA	2152 (49.85%)	574 (26.67%)	1578 (73.33%)
MVPA	2165 (50.15%)	712 (32.89%)	1453 (67.11%)
Race, *n* (%)			
Mexican American	640 (14.83%)	215 (33.59%)	425 (66.41%)
Other Hispanic	523 (12.11%)	173 (33.08%)	350 (66.92%)
Non-Hispanic White	1621 (37.55%)	546 (33.68%)	1075 (66.32%)
Non-Hispanic Black	978 (22.65%)	184 (18.81%)	794 (81.19%)
Other race	555 (12.86%)	168 (30.27%)	387 (69.73%)
Education level, *n* (%)			
< high school	1150 (26.64%)	328 (28.52%)	822 (71.48%)
High school graduate or equivalent	1011 (23.42%)	271 (26.80%)	740 (73.20%)
Some college or AA degree	1208 (27.98%)	338 (27.98%)	870 (72.02%)
> college graduate	948 (21.96%)	349 (36.81%)	599 (63.19%)
Marital status, *n* (%)			
Living without spouse	1721 (39.87%)	456 (26.50%)	1265 (73.50%)
Living with spouse	2596 (60.13%)	830 (31.97%)	1766 (68.03%)
Family PIR, *n* (%)			
< 1.0	755 (17.49%)	185 (24.50%)	570 (75.50%)
1.0-2.0	1134 (26.27%)	318 (28.04%)	816 (71.96%)
2.0-4.0	1004 (23.26%)	301 (29.98%)	703 (70.02%)
≥ 4.0	949 (21.98%)	342 (36.04%)	607 (63.96%)
missing	475 (11.00%)	140 (29.47%)	335 (70.53%)

Abbreviations as in Table S3.

Table S6. General characteristics of male participants.

**Variables**	**Overall** (*n* = 6929)	**Hypertension or not**	
		No (*n* = 3949)	Yes (*n* = 2980)
Age (years), mean ± SD	48.23 ± 16.44	42.18 ± 15.46	56.25 ± 14.10
SE, *n* (%)			
No	4604 (66.45%)	2576 (55.95%)	2028 (44.05%)
Yes	2325 (33.55%)	1373 (59.05%)	952 (40.95%)
PA, *n* (%)			
INPA	2191 (31.62%)	1052 (48.01%)	1139 (51.99%)
MVPA	4738 (68.38%)	2897 (61.14%)	1841 (38.86%)
Race, *n* (%)			
Mexican American	1066 (15.38%)	675 (63.32%)	391 (36.68%)
Other Hispanic	698 (10.07%)	424 (60.74%)	274 (39.26%)
Non-Hispanic White	2495 (36.01%)	1411 (56.55%)	1084 (43.45%)
Non-Hispanic Black	1478 (21.33%)	689 (46.62%)	789 (53.38%)
Other race	1192 (17.20%)	750 (62.92%)	442 (37.08%)
Education level, *n* (%)			
< high school	1563 (22.56%)	883 (56.49%)	680 (43.51%)
High school graduate or equivalent	1666 (24.04%)	912 (54.74%)	754 (45.26%)
Some college or AA degree	1983 (28.62%)	1108 (55.87%)	875 (44.13%)
> college graduate	1717 (24.78%)	1046 (60.92%)	671 (39.08%)
Marital status, *n* (%)			
Living without spouse	2401 (34.65%)	1427 (59.43%)	974 (40.57%)
Living with spouse	4528 (65.35%)	2522 (55.70%)	2006 (44.30%)
Family PIR, *n* (%)			
< 1.0	1224 (17.66%)	692 (56.54%)	532 (43.46%)
1.0-2.0	1681 (24.26%)	947 (56.34%)	734 (43.66%)
2.0-4.0	1676 (24.19%)	961 (57.34%)	715 (42.66%)
≥ 4.0	1676 (24.19%)	959 (57.22%)	717 (42.78%)
missing	672 (9.70%)	390 (58.04%)	282 (41.96%)

Abbreviations as in Table S3.

Table S7. General characteristics of female participants.

Variables	Overall (*n* = 7527)	Hypertension or not	
		No (*n* = 4495)	Yes (*n* = 3032)
Age (years), mean ± SD	47.72 ± 16.13	41.05 ± 14.33	57.61 ± 13.33
SE, *n* (%)			
No	5938 (78.89%)	3574 (60.19%)	2364 (39.81%)
Yes	1589 (21.11%)	921 (57.96%)	668 (42.04%)
PA, *n* (%)			
INPA	3347 (44.47%)	1755 (52.44%)	1592 (47.56%)
MVPA	4180 (55.53%)	2740 (65.55%)	1440 (34.45%)
Race, *n* (%)			
Mexican American	1193 (15.85%)	781 (35.46%)	412 (64.54%)
Other Hispanic	888 (11.80%)	530 (59.68%)	358 (40.32%)
Non-Hispanic White	2577 (34.24%)	1581 (61.35%)	996 (38.65%)
Non-Hispanic Black	1628 (21.63%)	770 (47.30%)	858 (52.70%)
Other race	1241 (16.49%)	833 (67.12%)	408 (32.88%)
Education level, *n* (%)			
< high school	1511 (20.07%)	775 (51.29%)	736 (48.71%)
High school graduate or equivalent	1588 (21.10%)	886 (55.79%)	702 (44.21%)
Some college or AA degree	2548 (33.85%)	1519 (59.62%)	1029 (40.38%)
> college graduate	1880 (24.98%)	1315 (69.95%)	565 (30.05%)
Marital status, *n* (%)			
Living without spouse	3263 (43.35%)	1798 (55.10%)	1465 (44.90%)
Living with spouse	4264 (56.65%)	2697 (63.25%)	1567 (36.75%)
Family PIR, *n* (%)			
< 1.0	1562 (20.75%)	900 (57.62%)	662 (42.38%)
1.0-2.0	1793 (23.82%)	1023 (57.06%)	770 (42.94%)
2.0-4.0	1784 (23.70%)	1101 (61.72%)	683 (38.28%)
≥ 4.0	1635 (21.72%)	1057 (64.65%)	578 (35.35%)
missing	753 (10.00%)	414 (54.98%)	339 (45.02%)

Abbreviations as in Table S3.

Table S8. ORs for hypertension according to SE and MVPA by age and sex.

Subgroup	*n*	*β*	Wald	OR	95% CI	*p*
SE						
Age						
20-39	5018	0.302	5.759	1.353	1.045-1.752	**0.024**
40-59	5121	0.181	3.318	1.199	0.977-1.471	0.080
60-79	4317	-0.155	1.603	0.856	0.666-1.101	0.216
Sex						
Male	6929	0.136	2.505	1.146	0.961-1.367	0.125
Female	7527	0.231	5.501	1.260	1.030-1.541	**0.026**
MVPA						
Age						
20-39	5018	-0.131	1.162	0.877	0.684-1.126	0.291
40-59	5121	-0.332	13.694	0.717	0.597-0.862	**< 0.001**
60-79	4317	-0.373	9.736	0.688	0.539-0.880	**0.004**
Sex						
Male	6929	-0.314	15.015	0.730	0.618-0.862	**< 0.001**
Female	7527	-0.272	10.706	0.762	0.643-0.903	**0.003**

Notes: Models were adjusted for age, sex, race, education level, marital status, family PIR, survey wave, and another exposure assessment. Abbreviations as in Table S2.

Table S9. The results of sensitive analyses.

Variables	*β*	Wald	OR	95% CI	*p*
SE					
Model A (*n* = 13031)	0.163	6.396	1.177	1.031-1.343	**0.017**
Model B (*n* = 14456)	0.005	4.657	1.005	1.000-1.011	**0.040**
MVPA					
Model A (*n* = 13031)	-0.347	27.092	0.706	0.616-0.810	**< 0.001**
Model B (*n* = 14456)	-0.291	25.324	0.747	0.664-0.842	**< 0.001**

Notes: Model A: delete the participants whose family PIR variable was missing. Model B: ORs for hypertension according to per IQR increase of serum cotinine concentration. Above models were adjusted for age, sex, race, education level, marital status, family PIR, survey wave, and another exposure assessment. Abbreviations: IQR, interquartile range; other abbreviations as in Table S2.

IQR increase: serum cotinine, 12.99 ng/mL.

Table S10. Joint effects between SE and PA on hypertension.

Variables	INPA		MVPA	
	OR (95% CI)	*p*	OR (95% CI)	*p*
Overall (*n* = 14456), *p* for interaction = 0.077				
Non-SE	1.000 (Reference)	1.000	0.740 (0.654-0.837)	**< 0.001**
SE	1.148 (0.934-1.409)	0.180	0.880 (0.747-1.037)	0.122
Age				
< 60 years (*n* = 10139), *p* for interaction = **0.043**				
Non-SE	1.000 (Reference)	1.000	0.739 (0.634-0.861)	**< 0.001**
SE	1.175 (0.937-1.472)	0.155	0.974 (0.820-1.157)	0.758

Notes: Models were adjusted for age, sex, race, education level, marital status, family PIR, and survey wave. Abbreviations as in Table S3.

Table S11. General characteristics of participants aged less than 60 years.

Variables	Overall (*n* = 10139)	Hypertension or not	
		No (*n* = 7158)	Yes (*n* = 2981)
Age (years), mean ± SD	39.61 ± 11.46	37.05 ± 11.03	45.76 ± 10.06
Sex, *n* (%)			
Male	3386 (33.40%)	2233 (65.95%)	1153 (34.05%)
Female	6753 (66.60%)	4925 (72.93%)	1828 (27.07%)
SE, *n* (%)			
No	7101 (70.04%)	5129 (72.23%)	1972 (27.77%)
Yes	3038 (29.96%)	2029 (66.79%)	1009 (33.21%)
PA, *n* (%)			
INPA	3386 (33.40%)	2233 (65.95%)	1153 (34.05%)
MVPA	6753 (66.60%)	4925 (72.93%)	1828 (27.07%)
Race, *n* (%)			
Mexican American	1619 (15.97%)	1241 (76.65%)	378 (23.35%)
Other Hispanic	1063 (10.48%)	781 (73.47%)	282 (26.53%)
Non-Hispanic White	3451 (34.04%)	2446 (70.88%)	1005 (29.12%)
Non-Hispanic Black	2128 (20.99%)	1275 (59.92%)	853 (40.08%)
Other race	1878 (18.52%)	1415 (75.35%)	463 (24.65%)
Education level, *n* (%)			
< high school	1924 (18.98%)	1330 (69.13%)	594 (30.87%)
High school graduate or equivalent	2243 (22.12%)	1527 (68.08%)	716 (31.92%)
Some college or AA degree	3323 (32.77%)	2289 (68.88%)	1034 (31.12%)
> college graduate	2649 (26.13%)	2012 (75.95%)	637 (24.05%)
Marital status, *n* (%)			
L`iving without spouse	3943 (38.89%)	2769 (70.22%)	1174 (29.78%)
Living with spouse	6196 (61.11%)	4389 (70.84%)	1807 (29.16%)
Family PIR, *n* (%)			
< 1.0	2031 (20.03%)	1407 (69.28%)	624 (30.72%)
1.0-2.0	2340 (23.08%)	1652 (70.60%)	688 (29.40%)
2.0-4.0	2456 (24.22%)	1761 (71.70%)	695 (28.30%)
≥ 4.0	2362 (23.30%)	1674 (70.87%)	688 (29.13%)
missing	950 (9.37%)	664 (69.89%)	286 (30.11%)

Abbreviations as in Table S3.
